# Integrin ανβ5 in vitro inhibition limits pro-fibrotic response in cardiac fibroblasts of spontaneously hypertensive rats

**DOI:** 10.1186/s12967-018-1730-1

**Published:** 2018-12-12

**Authors:** Gianluca Lorenzo Perrucci, Veronica Antonietta Barbagallo, Maria Corlianò, Delfina Tosi, Rosaria Santoro, Patrizia Nigro, Paolo Poggio, Gaetano Bulfamante, Federico Lombardi, Giulio Pompilio

**Affiliations:** 1 Unità di Biologia Vascolare e Medicina Rigenerativa, Dipartimento di Scienze Cliniche e di Comunità, Università degli Studi di Milano, via Festa del Perdono 7, Milan, Italy; 2 Unità di Biologia Vascolare e Medicina Rigenerativa, Centro Cardiologico Monzino IRCCS, via Carlo Parea 4, Milan, Italy; 3 Unità di Patologia, Dipartimento di Scienze della Salute, Università degli Studi di Milano, Ospedale San Paolo, via Antonio di Rudinì 8, Milan, Italy; 4 Unità per lo Studio di Patologie Aortiche, Valvolari e Coronariche, Centro Cardiologico Monzino IRCCS, via Carlo Parea 4, Milan, Italy; 5 Unità di Cardiologia, Fondazione IRCCS Ca’ Granda Ospedale Maggiore Policlinico, via Francesco Sforza 35, Milan, Italy

**Keywords:** Hypertension, Myofibroblast, TGF-β1, Integrin ανβ5, Cilengitide

## Abstract

**Background:**

To date the TGF-β1 activation mediated by integrin ανβ5 during fibrosis is well-known. This process has been shown also in the heart, where cardiac fibroblasts (CF) differentiate into α-smooth muscle actin (α-SMA)-positive myofibroblasts (MyoFB). Here, we studied the effects on CF, isolated by spontaneously hypertensive rats (SHR), of integrin ανβ5 inhibition in MyoFB differentiation.

**Methods:**

Staining and immunohistochemistry were performed on rat cardiac tissue. CF were isolated by enzymatic digestion from SHR (SHR-CF) and normotensive WKY (WKY-CF) rat hearts and then treated for in vitro evaluation.

**Results:**

SHR heart tissues revealed a higher TGF-β1 expression vs. WKY samples. SHR-CF showed an enhanced SMAD2/3 activation and an up-regulated expression of α-SMA, a typical MyoFB marker, especially after TGF-β1 treatment. Immunostaining on cardiac tissues revealed a higher expression of integrin ανβ5 in SHR vs. WKY rat hearts. In vitro results confirmed the up-regulation of integrin ανβ5 expression in SHR-CF at basal condition and after TGF-β1 treatment, in comparison with WKY-CF. Inhibition of integrin ανβ5 by cilengitide treatment led a decreased expression of ανβ5, collagen I, and α-SMA in SHR-CF vs. WKY-CF, resulting in a diminished differentiation of CF into MyoFB. Taking together, results suggested that SHR-CF are more susceptible to TGF-β1, showing an up-regulated activation of SMAD2/3 signaling, and an increased ανβ5, α-SMA, and collagen I expression. Hypertension stimulus promoted an up-regulation of integrin ανβ5 on SHR cardiac tissue and its in vitro inhibition reverted pro-fibrotic events of SHR-CF.

**Conclusion:**

Inhibition of integrin ανβ5 exerted by cilengitide strongly diminished SHR-CF differentiation into detrimental MyoFB. So, integrin ανβ5 might be considered a novel therapeutic target and cilengitide an effective pharmacological tool to limit the progression of hypertension-induced cardiac fibrosis.

**Electronic supplementary material:**

The online version of this article (10.1186/s12967-018-1730-1) contains supplementary material, which is available to authorized users.

## Background

Arterial hypertension is one of the main cause of cardiac remodelling, which commonly culminates in left ventricular hypertrophy (LVH) in about 15–20% of hypertensive patients [1]. Although current anti-hypertensive drugs reduce arterial blood pressure, these treatments are partially effective in reducing or reverting the hypertensive-derived cardiac fibrosis. Thus, the identification of further targets is extremely relevant in this pathological context, in order to define novel and proper therapeutic strategies.

In the last 20 years, the transforming growth factor-β1 (TGF-β1) has emerged as a crucial mediator of the local cardiac remodelling, not only in hypertensive pathological scenario [2], but also in other cardiac disease [3]. In particular, active TGF-β1 enhances the differentiation of fibroblasts into contractile myofibroblasts (MyoFB), by promoting the expression of α-smooth muscle actin (α-SMA) [4, 5], and the secretion of detrimental pro-fibrotic collagen in extracellular matrix (ECM) [6]. Thus, persistent activation of MyoFB results in the formation of scars, which determines an increased ECM stiffness and leading to an impaired heart function [7].

Although the TGF-β1 activation models are numerous, among these the integrin-mediated mechanotransduction of TGF-β1 release from its latent complex in ECM is gaining in last years a strong relief [8, 9]. This model has been primarily described starting from studies on lung and skin fibrotic disease [10–12]. In fact, it has been reported in these pathologies that several integrins are able to bind the ECM component of TGF-β1 latent complex and to release TGF-β1 by mechanical stretching [13–16].

Among these integrins, the integrin ανβ5, constitutively expressed in normal cardiac tissue, has been shown to mediate the adhesion of rat fibroblasts to vitronectin in ECM [17]. In addition, Sarrazy et al. have recently shown that human cardiac fibroblasts (CF) are able to differentiate into contractile MyoFB by integrin-mediated TGF-β1 activation in a pig model of LVH [18].

Although in other pathological contexts (i.e. glioblastoma, pancreatic and breast cancer, idiopathic pulmonary fibrosis) the integrin-mediated TGF-β1 activation is a well-known pharmacological axis and several compounds have been already included in clinical trials [19], the involvement of this molecular mechanism, as well as its inhibition, in hypertension-derived cardiac fibrosis has not been yet evaluated. In depth, cilengitide is an effective ανβ5 integrin inhibitor, which has been studied in the last 10 years as therapeutic compound in a series of pre-clinical and clinical trials [20–24].

Thus, aims of this study are the investigation in hypertension context of TGF-β1 signaling activation in terms of integrin ανβ5 expression, both in heart tissue and in CF isolated from hypertensive rats, and the in vitro modulation of this axis exerted by specific integrin inhibitor.

## Methods

### Animals

8-week-old male normotensive Wistar Kyoto rats (WKY, *n *= 10) and Spontaneously Hypertensive rats (SHR, *n *= 10) were housed in collective cages under 14–10 h light/dark cycles in a temperature-controlled room (22 °C). In order to confirm hypertension in the SHR group, diastolic (DBP) and systolic (SBP) blood pressures were measured at the beginning of the experiments by tail-cuff plethysmography (BP-2000; Visitech Systems, Apex, North Carolina, USA). Mean blood pressure (MBP) was calculated as previously described [25]. DBP, SBP, and MBP values were reported in Table 1.

**Table 1:** Diastolic, systolic, and mean arterial blood pressure in WKY and SHR rat groups

	WKY (*n *= 10)	SHR (*n *= 10)	
DBP (mmHg)	92 (± 10)	123 (± 14)	***
SPB (mmHg)	107(± 6)	143 (± 7)	***
MBP (mmHg)	97 (± 9)	97 (± 8)	***

Blood pressure values are expressed as mean ± SD, *n *= 10/group

*DBP* diastolic blood pressure, *SBP* systolic blood pressure, *MBP* mean blood pressure

Student’s *t*-test: ***p < 0.0001

### Tissue staining and immunohistochemistry

WKY and SHR (*n *= 5/group) were anaesthetized and perfused with normal saline. Hearts were extracted, washed in PBS, fixed with 10% phosphate-buffered formalin and paraffin embedded, then 5 µm-thick sections were cut from each sample. Masson’s trichrome staining kit was used to perform the assay (Bio-Optica, #04-010802). For immunohistochemistry assays, formaldehyde-fixed paraffin sections were kept 35 min at 97.5 °C in 9 mM sodium citrate pH 6.0. Endogenous peroxidase activity was quenched with 3% H_2_O_2_ for 10 min; incubation of primary antibodies was performed overnight (O/N). Staining was performed with 3,3-diaminobenzidine (DAB) as a chromogen. Slides were immunostained in the same batch, including negative controls lacking the primary antibody. Antibodies raised against TGF-β1 (AbCam, ab64715, clone 9016) and ανβ5 (AbCam, ab179475, clone EPR16800) were used. As a negative control, species- and isotype-matched IgGs were incubated in place of the primary antibodies. Serial sections derive from comparable areas of the left rat heart ventricle, and were viewed with AxioSkop microscope equipped with AxioCam camera (Carl Zeiss) and analyzed with AxioVision 4.7 software (Carl Zeiss). The positive areas for TGF-β1 and ανβ5 on heart samples were normalized to the section area, calculated with AxioVision 4.7 software (Carl Zeiss).

### Cell isolation and culture

WKY and SHR (*n *= 5) were anaesthetized by 2% isoflurane and euthanized by cervical dislocation. Primary CF were isolated from rat whole hearts by the following protocol. Hearts were extracted with sterile pliers and placed in tubes with sterile DMEM High Glucose, with 1% penicillin/streptomycin, 0.5% gentamicin, and 1% amphotericin B (EuroClone). Cardiac tissue was transferred onto a tissue culture glass dish and minced using disposable sterile scalpels. Tissue fragments were incubated at 37 °C for 15 min in 10 ml of digestion buffer, composed by phosphate buffer saline (PBS, Euroclone), 1% penicillin/streptomycin, 1% Amphotericin B, and Liberase TH Research Grade Blendzyme (Roche). After washing 3 times with PBS, digested tissue fragments were cultured on vitronectin-coated dishes at 37 °C, 5% CO_2_ in DMEM High Glucose (EuroClone) supplemented with 15% Fetal Bovine Serum (FBS, EuroClone), 2 mM l-glutamine, 200 U/ml penicillin, 200 μg/ml streptomycin, and 0.5% Amphotericin B. For the signaling experiments, CF were serum starved and treated with 5 ng/ml TGF-β1 (Peprotech) for 30 min, while, for the other experiments, cell were treated with 5 ng/ml TGF-β1 and 0.5 μM cilengitide (MedChem Express) for 48 h. For stiffness-dependent experiments, two substrates of known stiffness (4.1 kPa, 30 kPa) were produced by varying the content of acrylamide and the ratio acrylamide/bisacrylamide, according to a previously published method [26]. Briefly, small drops (70 μl) of polyacrylamide solution were deposited onto glass slides (30 mm diameter); glass coverslips, previously treated with Surfacil (Pierce) were placed on the top of the solution drops and kept under nitrogen flow, until thin uniform polyacrylamide gels were formed. Finally, polyacrylamide gels surface was chemically activated (Sulfo-SANPAH-Pierce), and coated by vitronectin solution (ThermoFisher, #A14700) incubation O/N at 4 °C. Substrate sterilization was performed by 30 min UV (254 nm) light exposure.

### ImageStreamX assay

Nuclear translocation of active form of SMAD2 in CF after TGF-β1 treatment was evaluated by ImageStreamX flow cytometer, a flow cytometric technique combined with a fluorescence microscope (ImageStreamX Mark II, Amnis). WKY-CF and SHR-CF were serum starved and treated for 3 h with 5 ng/ml TGF-β1 (Peprotech). Cells were detached from Petri dishes by using a nonenzymatic method (TripLE™ Select, Gibco), then, in order to allow primary antibody hybridization, CF were fixed, permeabilized by using BD Cytofix/Cytoperm kit (BD Pharmingen, 554714, clone EP567Y), and then incubated in the dark for 15 min with 0.5 μg/ml anti-SMAD2 primary antibody conjugated with FITC (Abcam, ab196320). Each sample was then washed with 1 ml of washing buffer and centrifuged for 10 min at 400×*g* to remove unbound antibody. Cells were resuspended in 100 μl of FACS buffer, composed by PBS supplemented with 0.1% BSA (Gibco) and 5 mM EDTA (Gibco), incubated with 2.5 μM nuclei fluorescent staining DRAQ5 (Abcam, ab108410), and analyzed. Instrument and INSPIRE software were set up as follows: channel (Ch) 01 for brightfield, Ch02 for FITC fluorescence intensity, Ch05 for APC fluorescence intensity, and Ch06 for side-scatter intensity. All samples were acquired with a magnification of 40× at a low flow rate (high sensitivity), and 488 nm, 630 nm, and 785 nm lasers were activated for FITC fluorescence, APC fluorescence, and side-scatter intensity, respectively. CF were gated on a dot plot reporting area (“x” axis) and aspect ratio (“y” axis) to eliminate cell clumps. A total of 10,000 events in the CF gated area were acquired. Image analysis was performed using the IDEAS image software. The degree of fluorescence relative to SMAD2-FITC staining was quantified using the Intensity_MC_Ch02, whereas DRAQ5 staining was quantified using the Intensity_MC_Ch05. To evaluate FITC-APC overlapping signal, indicating the nuclear translocation of SMAD2, a Similarity Dilate index analysis on Intensity_MC_Ch02 and Intensity_MC_Ch05 was performed. In this context, the Similarity Dilate index expresses the number of events (cells) in which FITC signal (SMAD2) is co-localized with the APC signal (DRAQ5).

### mRNA extraction and qRT-PCR

WKY-CF and SHR-CF were cultured and contemporary treated with 5 ng/ml recombinant TGF-β1 and/or 0.5 μM cilengitide for 48 h. RNA was isolated with a Total RNA Purification kit (Norgen Biotek corp.). RNA quantification was determined with Spectrophotometer ND-1000 (NanoDrop^®^, EuroClone). Reverse transcription was conducted with the SuperScript III (ThermoFisher, #18080093) following the manufacturer’s instructions. qRT-PCR was performed on the iQ™ SYBR Green Super Mix (Bio-Rad, #1725125). 5 ng of cDNA were used to quantify the expression of the following genes: *acta2* (FW: TGCCATGTATGTGGCTATTCA; RV: ACCAGTTGTACGTCCAGAAGC), *itgav* (FW: TCGCAGGGCTCAACATATG; RV: CTCTCAATCTCACCTCCACAG), and *tgfb1* (FW: ATGACATGAACCGACCCTTC; RV: GATCCACTTCCAACCCAGG). All reactions were performed in a 96-well format in the iQ5™ (Bio-Rad). The relative quantities of specific mRNAs were obtained with the use of the comparative Ct method and were normalized to GAPDH gene (FW: TGAAGGTCGGTGTGAACGG; RV: TCAATGAAGGGGTCGTTGAT).

### Western blot analysis

WKY-CF and SHR-CF were contemporary treated with 5 ng/ml recombinant TGF-β1 and/or 0.5 μM cilengitide for 48 h, and lysed in cell lysis buffer (Cell Signaling Technology, #9803) supplemented with protease and phosphatase inhibitor cocktails (Sigma-Aldrich). The same lysis buffer solution was used for total protein tissue extracts. Total cell and tissue proteins were subjected to SDS-PAGE and transferred onto a nitrocellulose membrane. The membranes were blocked for 1 h at room temperature in 5% non-fat dry milk in wash buffer (Tris Buffer Sulfate 1×, 0.1% Tween 20) and then incubated O/N at 4 °C with the appropriate primary antibody. The primary antibodies were specific for TGF-β1 (AbCam, ab64715, clone 9016), phospho-SMAD2 (Ser465/467)/SMAD3 (Ser423/425) (Cell Signaling, #8828, clone D27F4), SMAD2/3 (Cell Signaling, #3102), α-SMA (Merck Millipore, CBL171, clone ASM-1), ανβ3 (AbCam, ab7166, clone BV3), ανβ5 (AbCam, ab179475, clone EPR16800), and collagen I (AbCam, ab34710). The membranes were incubated at room temperature with peroxidase-conjugated secondary antibodies for 1 h. Signals were visualized using enhanced chemiluminescence Western blotting detection system (GE Healthcare). Proteins were normalized according to β-tubulin (Sigma-Aldrich, T9026, clone DM1A). Images were acquired with Alliance Mini 2M (UVITec Cambridge) and the densitometric analysis of membranes was performed using the Alliance Mini 4 16.07 software (UVITec Cambridge).

### TGF-β1 levels in conditioned medium of CF

WKY-CF and SHR-CF were treated with 0.5 μM cilengitide for 48 h, then supernatant conditioned media were collected and stored. TGF-β1 levels in conditioned medium were detected with an ELISA kit (LSBio, LS-F12740) following the manufacturer’s instructions.

### Immunofluorescence

WKY-CF and SHR-CF were plated on Chamber Slides (Nunc) and placed in growth for 24 h with 95% humidity and 5% CO_2_. CF were treated as previously described for 48 h. Then, slides were rinsed with PBS solution and soaked for about 15 min in a solution of 4% Paraformaldehyde (PFA). The primary unconjugated antibody raised against α-SMA (Merck Millipore, CBL171, clone ASM-1) was incubated O/N at 4 °C. The goat anti-mouse IgG1 secondary antibody conjugated with AlexaFluor488 (ThermoFisher, #A-21121) was incubated for 1 h at room temperature. As a negative control, species- and isotype-matched IgGs were incubated in place of the primary antibodies. Slides were viewed with Apotome microscope equipped with AxioCam camera (Carl Zeiss) and analyzed with AxioVision 4.7 software (Carl Zeiss).

### Statistical analyses

Quantitative results are expressed as mean ± SD. Statistical significance was evaluated with GraphPad Prism 5. Variables were analyzed by Student’s *t*-test and 2-way ANOVA, followed by a post hoc analysis using the Bonferroni post-test. A value of *p* ≤ 0.05 was deemed statistically significant.

## Results

### Hypertensive rat hearts show higher fibrosis and TGF-β1 expression than normotensive controls

**Fig. 1:**
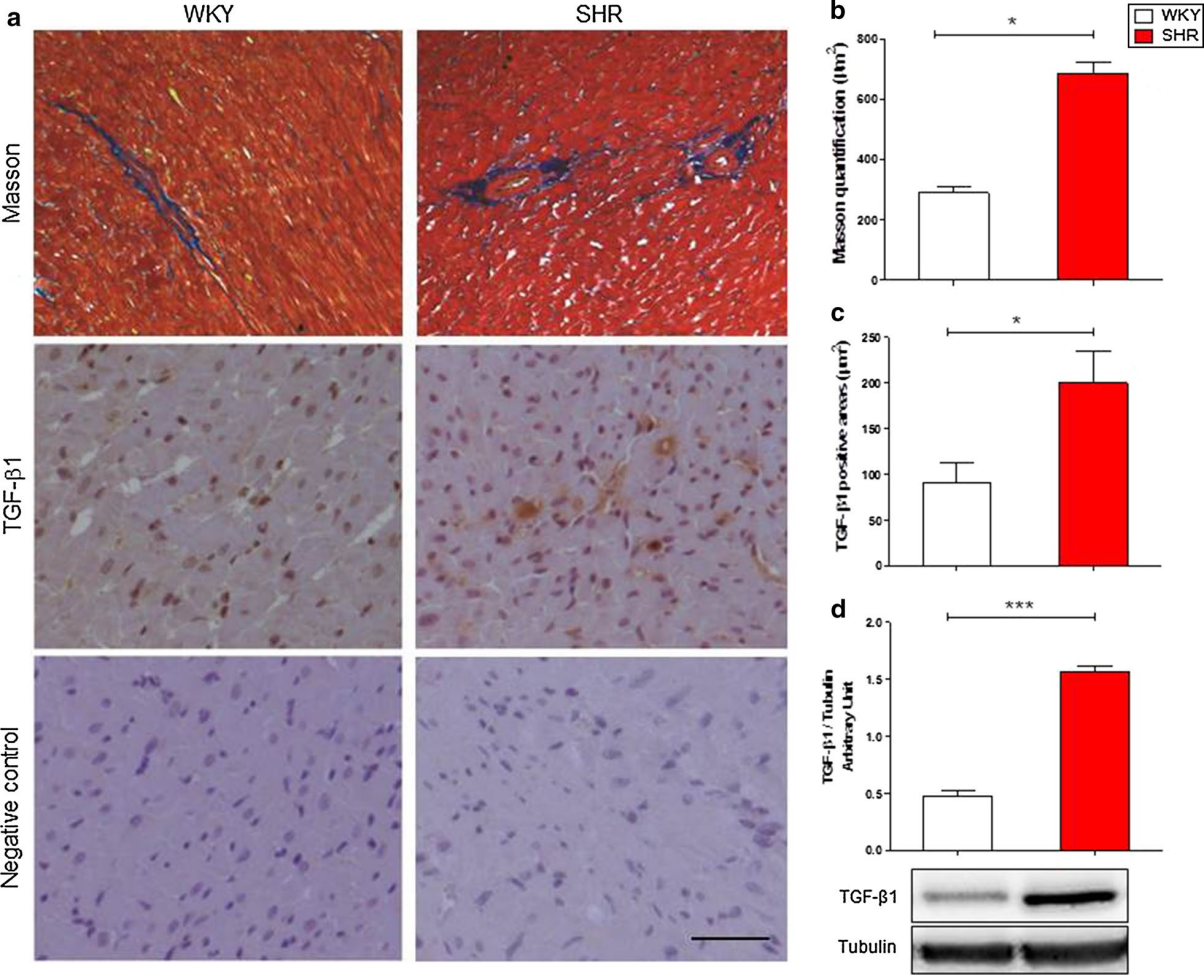
Collagen deposition and TGF-β1 expression are higher in SHR than WKY rat cardiac tissue. **a** Representative images of Masson’s trichrome staining, TGF-β1 immunostaining and relative secondary antibody negative control, on normotensive (WKY) and hypertensive (SHR) rat heart tissue samples. Quantification of collagen fibres (**b** blue signal in Masson’s trichrome staining) and TGF-β1 posive areas (**c** brown signal in immunohistochemistry) in WKY (white bars) and SHR (red bars) left ventricle samples. All histological quantifications are expressed as mean ± SD, *n *= 5/group. Scale bars = 100 μm. **d** TGF-β1 expression levels in WKY and SHR heart tissue protein extracts. Western blot quantification data are expressed as mean ± SD after β-tubulin normalization, *n *= 5/group. Student’s *t*-test: *p < 0.05; ***p < 0.0001

It is well known that a common feature of hypertension is LVH, mainly characterized by the cardiac tissue remodelling, in which TGF-β1 plays a major role as molecular mediator of ECM alterations [2, 27]. For all these reasons, a Masson’s trichrome staining and an immunohistochemistry assay for TGF-β1 were performed in order to detect both collagen deposition and TGF-β1 expression on heart sections of SHR (*n *= 5) and WKY (*n *= 5) rats. Consistently, as highlighted in Fig. 1, cardiac tissues of hypertensive rats (SHR) showed a higher collagen deposition in perivascular region (Fig. 1a, b), together with an augmented TGF-β1 expression in comparison with normotensive (WKY) controls (Fig. 1a, c). To further confirm this immunohistological result, a Western bot analysis was performed to evaluate in-depth the TGF-β1 expression level in total protein extracts from WKY and SHR heart tissue (Fig. 1d). Figure 1d confirmed the result previously showed in Fig. 1a, c.

Thus, hypertensive stimulus leads to a higher collagen and TGF-β1 expression on cardiac tissue.

### SMAD2/3 signaling activation and α-SMA expression levels are higher in SHR-CF after TGF-β1 treatment

**Fig. 2:**
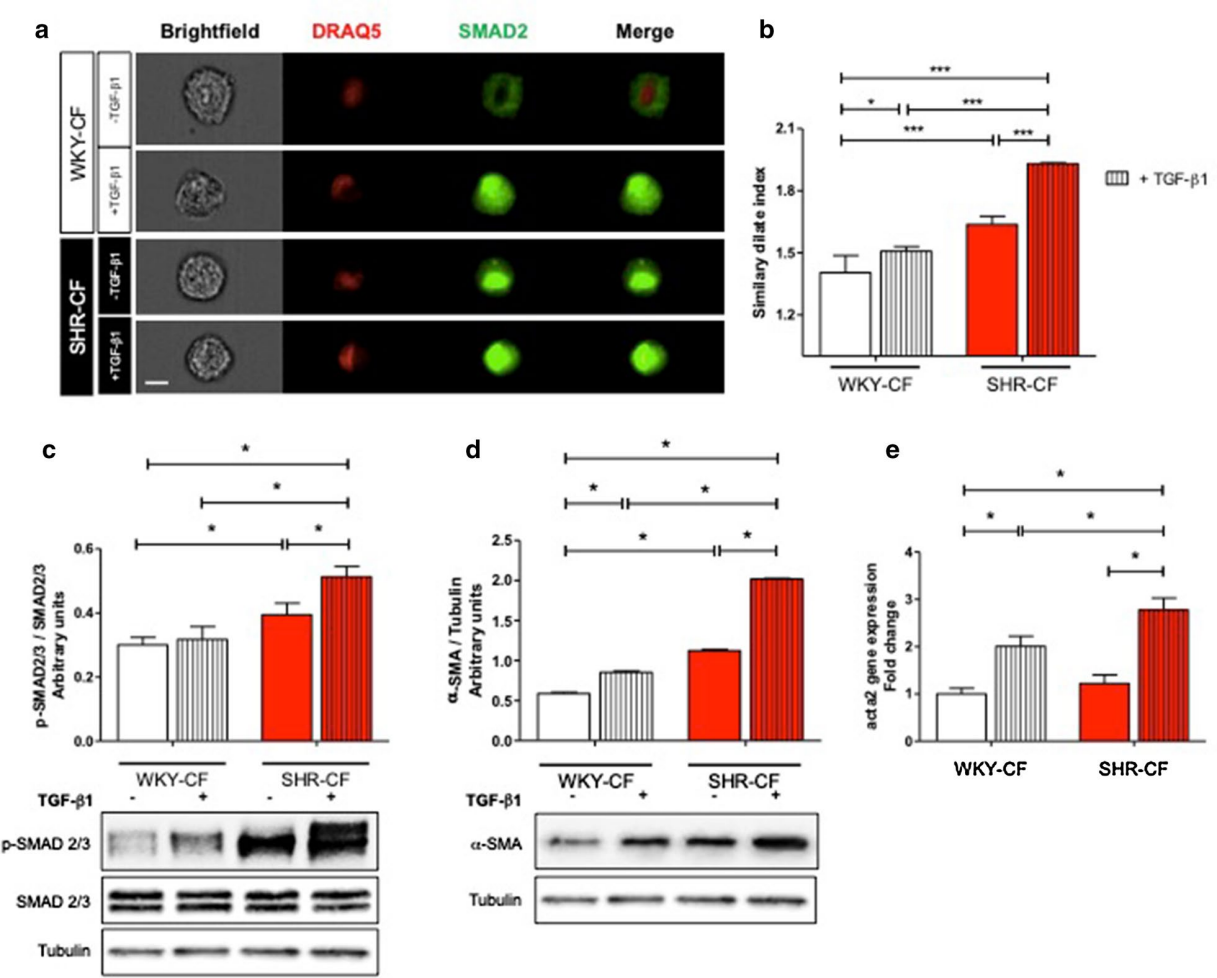
TGF-β1 treatment increases SMAD2/3 signaling activation and α-SMA expression levels more in SHR-CF than in WKY-CF. **a** Results obtained by ImageStreamX (IMX) technology, on SMAD2 nuclear translocation in WKY- and SHR-CF before and after 3 h of TGF-β1 treatment. IMX microscopy images were obtained on fixed and permeabilized CF, hybridized with anti-SMAD2-FITC antibody (green), and stained with DRAQ5 for the nucleus (red). Merged signal (yellow) indicates SMAD2 signal after nuclear translocation. **b** IMX fluorescence-activated cell sorting analysis on data collected from 10,000 events/sample. Bar graph indicates the similarity dilate index of WKY-CF (white bars) and SHR-CF (red bar), without and with TGF-β1 treatment. Scale bar = 10 μm. **c** Phospho-SMAD2/3 and total SMAD2/3 expression levels in WKY- and SHR-CF after 30 min of treatment with 5 ng/ml of recombinant TGF-β1. α-SMA protein (**d**) and gene (**e**) expression in WKY- and SHR-CF after treatment with 5 ng/ml TGF-β1. Western blot quantification data are expressed as mean ± SD after β-tubulin normalization, qRT-PCR data are expressed as fold ± SD normalized with GAPDH gene, *n *= 5/group. The experiments on cells were performed in triplicate. 2-way ANOVA with Bonferroni’s post-test: *p < 0.05, ***p < 0.001

To date, the detrimental role played by TGF-β1 on CF in the adverse hypertension-derived myocardial remodelling is well established [28]. Thus, to better investigate the molecular pathways specifically activated in these cells, CF were isolated from the heart of each rat strain (*n *= 5 WKY, *n *= 5 SHR) for in vitro treatments and subsequent evaluations. Firstly, a full characterization of CF was performed, to evaluate the fibroblastic nature of these cells. Immunofluorescence and FACS analyses revealed that isolated CF were mesenchymal vimentin-positive cells (Additional file 1: Figure S1A), negative for cardiomyocyte, endothelial, and inflammatory cell markers (Additional file 1: Figure S1B). Then, in order to in-depth investigate the activation of TGF-β1 pathway in terms of SMAD2/3 signaling, ImageStreamX and Western blot analyses were performed. Figure 2a, b revealed a significantly higher nuclear translocation of SMAD2/3, subsequent to its phosphorylation (shown in Fig. 2c), in SHR-CF when compared to WKY-CF. Moreover, this event was further emphasized in the same cells after 5 ng/ml TGF-β1 treatment. In addition, several studies in the last years well defined α-SMA as a downstream product of TGF-β1/SMAD signaling, as well as an essential mediator involved in the CF differentiation into MyoFB [29]. Figure 2d clearly showed, by Western blot analysis, a strong up-regulated α-SMA expression of SHR-CF in comparison with WKY-CF. In depth, SHR-CF showed a statistical increase of α-SMA protein expression at baseline after 48 h of culture when compared to untreated WKY-CF. Moreover, in both CF types 48 h of TGF-β1 treatment exacerbates the up-regulation of α-SMA, which resulted strongly increased specifically in SHR-CF. Similarly, the expression of *acta2* gene, encoding α-SMA protein, was statistically higher in SHR-CF after TGF-β1 treatment than in untreated CF of both rat strains and also in comparison with TGF-β1 treated WKY-CF (Fig. 2e).

All these data suggest that cultured SHR-CF (i) display a constitutively activated SMAD2/3 signaling together with a greater responsiveness to TGF-β1 and (ii) are more prone to differentiate into MyoFB by expressing a higher amount of α-SMA when compared to WKY-CF.

### ανβ5 gene and protein expression is up-regulated both in SHR cardiac tissue and SHR-CF

**Fig. 3:**
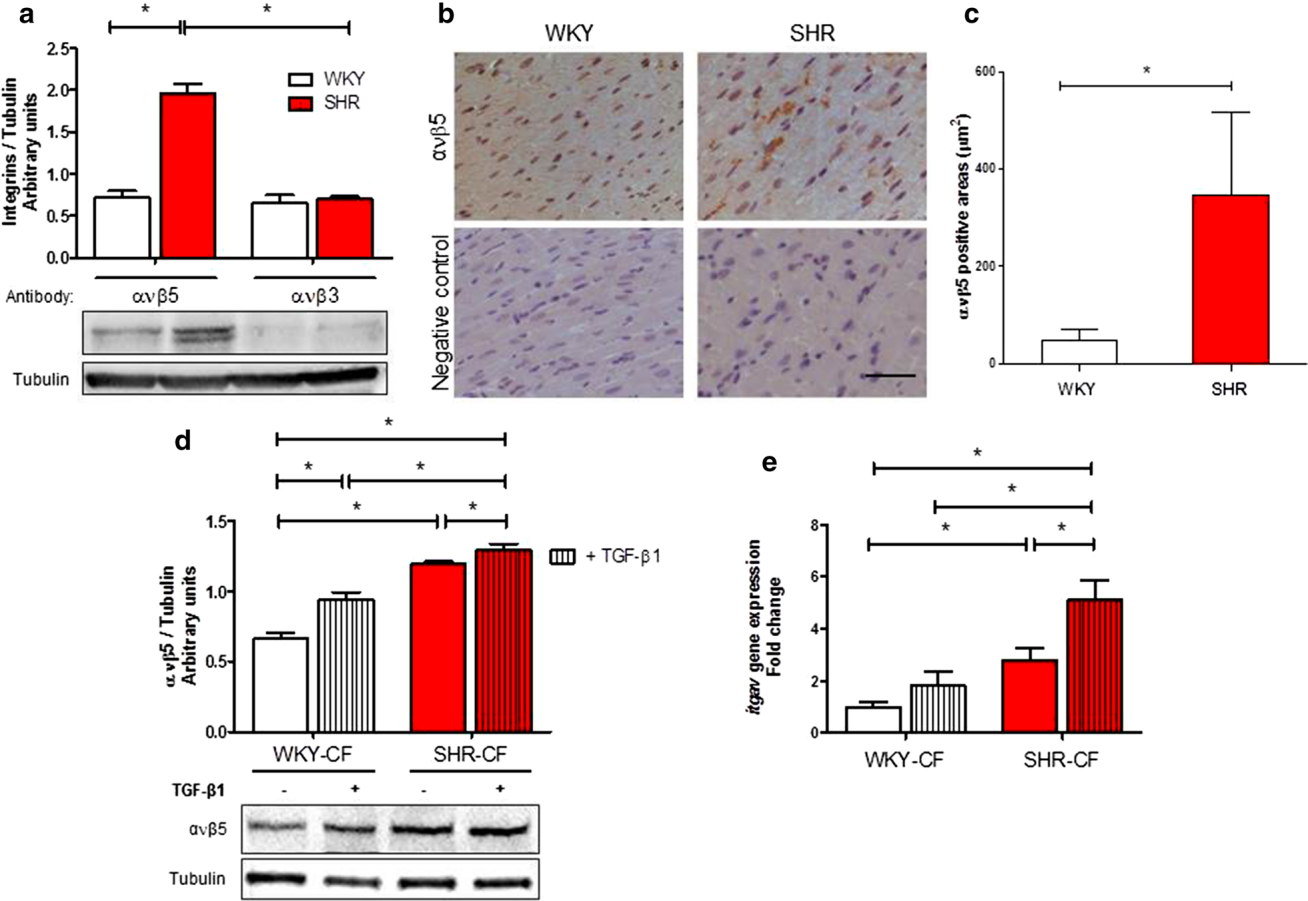
Integrin ανβ5 expression is higher both in SHR cardiac tissue and in SHR-CF than respective controls. **a** Integrin ανβ5 and ανβ3 expression levels in WKY (white bars) and SHR (red bars) heart tissue protein extracts. Western blot quantification data are expressed as mean ± SD after β-tubulin normalization, *n *= 5/group. Student’s *t*-test: *p < 0.05. **b**, **c** Integrin ανβ5 immunohistochemistry and relative secondary antibody negative control on normotensive (WKY) and hypertensive (SHR) rat heart sample (**b**). Integrin ανβ5 positive areas were quantified and normalized on whole sample area (**c**). Student’s *t*-test: *p < 0.05. Scale bar = 100 μm. Integrin ανβ5 protein (**d**) and *itgav* gene (**e**) expression in WKY- and SHR-CF after treatment with 5 ng/ml TGF-β1. Western blot quantification data are expressed as mean ± SD after β-tubulin normalization, qRT-PCR data are expressed as fold ± SD normalized with GAPDH, *n *= 5/group. The experiments on cells were performed in triplicate. 2-way ANOVA with Bonferroni’s post-test: *p < 0.05

Several previous data suggested a massive involvement of different component of integrin protein family in TGF-β1 activation by mechanotransduction mechanism, but little is still known about the effect of hypertension and TGF-β1 signaling in integrin expression on CF. Thus, we evaluated the expression of integrin ανβ3 and ανβ5 by Western blot on WKY and SHR rat heart tissue protein extract, to understand on which integrin deepen the in vitro investigations. The results of this assay, shown in Fig. 3a, clearly highlighted the higher expression of integrin ανβ5 in tissue samples than integrin ανβ3. In depth, the integrin ανβ5 expression resulted statistically higher in SHR than WKY cardiac tissue. The results collected by immunohistochemistry on tissue confirmed the higher expression of ανβ5 in SHR vs. WKY cardiac tissue (Fig. 3b, c). In order to evaluate the integrin ανβ5 expression levels also in vitro, SHR- and WKY-CF were treated with TGF-β1, then both Western blot and qRT-PCR assays were performed on protein and total RNA extracts, respectively. Integrin ανβ5 protein expression was statistically higher in SHR-CF after 48 h of TGF-β1 treatment in comparison with untreated SHR-CF and WKY-CF with and without TGF-β1 treatment (Fig. 3d). Real-time data demonstrated a statistically higher up-regulation of integrin αν subunit gene expression in untreated SHR-CF vs. WKY-CF; the statistical difference was, also in this case, enhanced by TGF-β1 treatment (Fig. 3e).

These findings suggest that hypertension is a sufficient stimulus in promoting integrin ανβ5 over-expression on cardiac tissue and also that the TGF-β1 is an effective mediator in stimulating up-regulation of ανβ5 transcription and production in SHR-CF.

### Specific ανβ5 blockade in vitro decreases typical MyoFB protein expression in SHR-CF

**Fig. 4:**
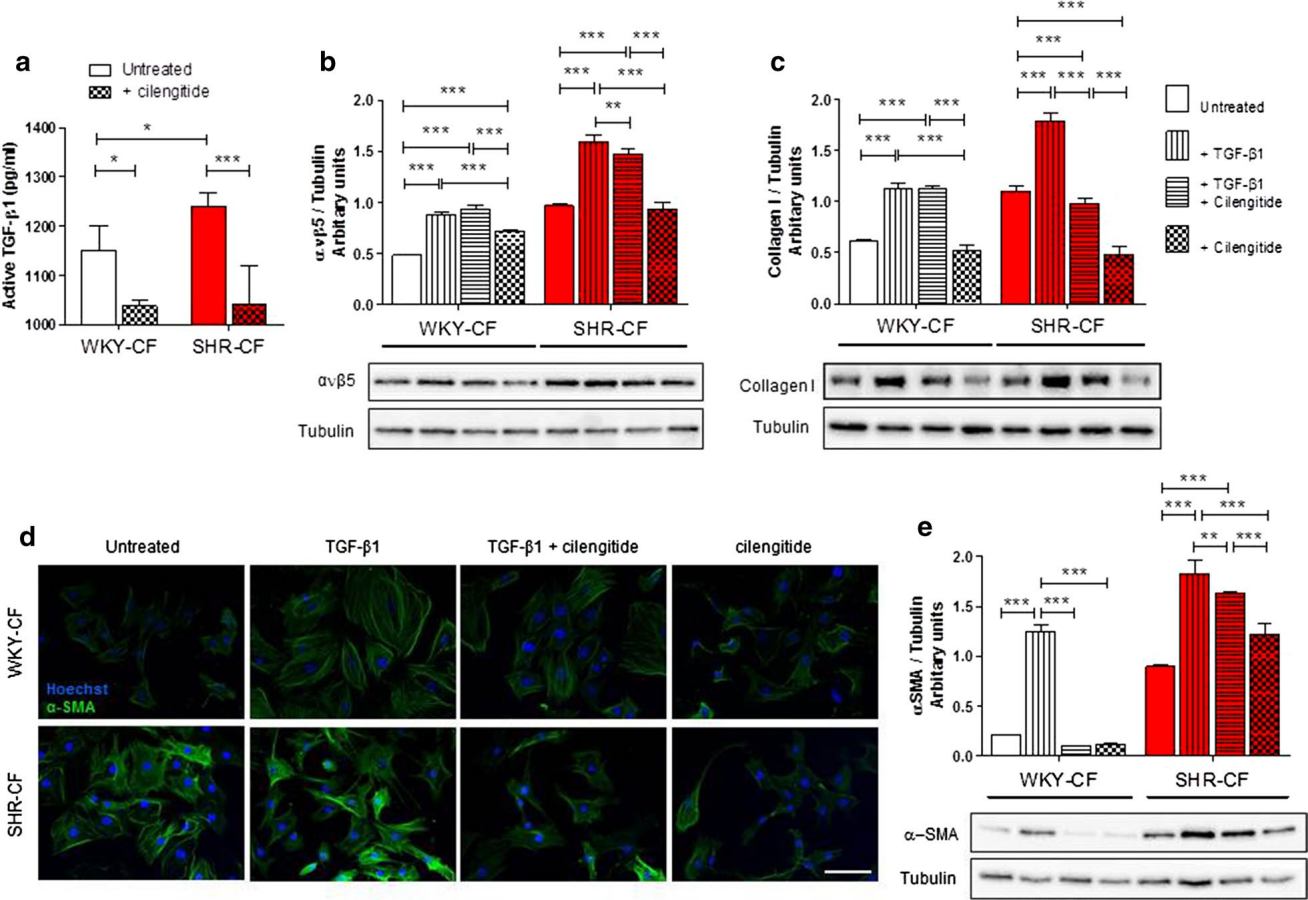
Integrin ανβ5 blockade in vitro decreases TGF-β1 release and protein production of typical MyoFB markers. **a** Secreted amount of active TGF-β1 in conditioned media of WKY- and SHR-CF after treatments with 0.5 μM cilengitide. ELISA data are expressed as mean ± SD, *n *= 5/group. The experiments on cell surnatants were performed in triplicate. Protein expression of integrin ανβ5 (**b**) and collagen I (**c**) in WKY- and SHR-CF after treatments with 5 ng/ml TGF-β1, TGF-β1 + 0.5 μM cilengitide, or cilengitide. **d** Representative images of immunofluorescence performed for α-SMA on WKY- and SHR-CF. Scale bar = 100 μm. **e** Protein expression of α-SMA in WKY- and SHR-CF after treatments with 5 ng/ml TGF-β1, TGF-β1 + 0.5 μM cilengitide, or cilengitide. All Western blot quantification data are expressed as mean ± SD after β-tubulin normalization. The experiments on cells were performed in triplicate, with a *n *= 5/group. 2-way ANOVA with Bonferroni’s post-test: *p < 0.05, **p < 0.01, ***p < 0.001

To evaluate whether integrin ανβ5 may be considered a putative pharmacological target in the adverse alteration of hypertensive-derived fibrosis, cilengitide was used as αν RGD integrin inhibitor for in vitro evaluations. To assess the cytotoxicity of this integrin inhibitor compound, a dose–response assay by MTT internalization (Additional file 1: Figure S2) was performed. Simultaneously, to evaluate the more effective concentration of cilengitide in vitro on CF, an ELISA assay (Fig. 4a) was further performed. Taken together, these results confirmed that cilengitide is not significantly toxic at the concentration of 0.5 μM. Furthermore, the integrin ανβ5 inhibitor at the same concentration is able in strongly decreasing the TGF-β1 release by SHR-CF in the conditioned medium. After that, as depicted in Fig. 4b, the protein expression of integrin ανβ5 was consistently and significantly down-regulated after 48 h of 0.5 μM cilengitide treatment when compared with TGF-β1 treatment in SHR-CF. It is noteworthy that integrin ανβ5 levels are statistically higher in SHR-CF vs. WKY-CF, but cilengitide treatment specifically rescued this protein expression in SHR-CF to the levels of untreated condition. In fact, it is also important to point out how cilengitide is more effective on SHR-CF than in WKY-CF in counteracting the TGF-β1 activity.

As previously reported, the main features of activated MyoFB are (i) the higher expression of α-SMA, which gives a contractile phenotype to these cells, and (ii) the greater production of collagen I and other ECM proteins, which strongly contribute to cardiac reactive fibrosis. Both these features were evaluated in our in vitro model: Western blot and immunofluorescence analyses were performed after 48 h of treatments. Specifically, integrin inhibition deeply diminished collagen I production in CF of both rat strains (Fig. 4c). Although the collagen I protein levels in SHR-CF were overall higher than those expressed by WKY-CF, in both CF cell types cilengitide treatment significantly down-regulated collagen I expression. Markedly, the collagen I level expressed by SHR-CF after cilengitide treatment was significantly lower than untreated SHR-CF, even reaching the expression levels of untreated WKY-CF. Similar results were obtained by immunofluorescence assays and Western blot analysis on α-SMA expression (Fig. 4d, e), where it was clearly showed an overall strong up-regulation of α-SMA in SHR-CF when compared with WKY-CF. In the context of immunofluorescence assay, it is important to point out that the inhibition of integrin mediated by cilengitide together with the repetitive cell washes, necessary in this technique, led to a lower number of attached cells on the chamber slides. For this reason and also in order to have a more quantifiable data, the Western blot assay was performed to provide the evidence on cilengitide effects on α-SMA.

As for collagen I, another pro-fibrotic mediator expression, such as laminin (Additional file 1: Figure S3A), resulted modulated by cilengitide, even in presence of TGF-β1. Real-time results on modulation of *tgfb1* gene, encoding TGF-β1, on CF after treatments were consistent with previously observed data on ανβ5, collagen I, and laminin protein assays. In fact, *tgfb1* was significantly down-regulated after 24 h of 0.5 μM cilengitide treatment when compared with TGF-β1 treatment, in both CF cell lines (Additional file 1: Figure S3B). It is important to highlight how *tgfb1* levels were statistically higher in SHR-CF vs. WKY-CF, but cilengitide treatment rescued this gene expression to that of untreated cells. Noteworthily, as for ανβ5, collagen I, and laminin levels, cilengitide is more effective on SHR-CF than in WKY-CF in fixing the detrimental effects of TGF-β1.

So, these data suggest that cilengitide activity strongly reduces the fibrotic progression in terms of MyoFB differentiation of CF, especially in SHR-CF.

### A higher stiffness of cell growth substrate exacerbates integrin ανβ5 and collagen I expression, as well as cilengitide effectiveness, in SHR-CF

**Fig. 5:**
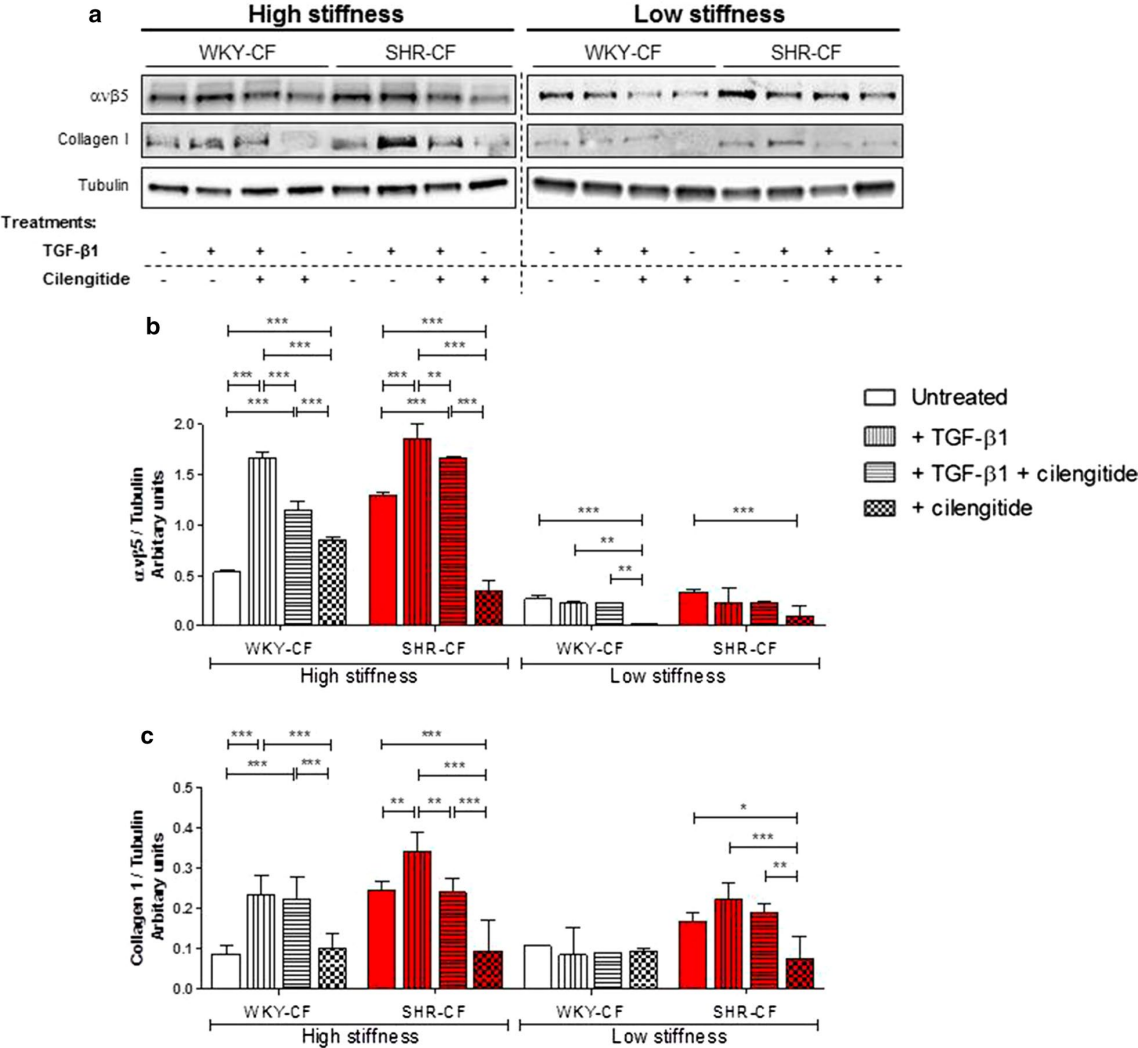
In vitro inhibition of integrin ανβ5 by cilengitide limits ανβ5 and collagen I expression more effectively on SHR-CF growing on a substrate with high stiffness. **a** Representative Western blot images of ανβ5 and collagen I protein expression in WKY- and SHR-CF cultured on substrates with two different stiffness (high and low) and contemporary treated with 5 ng/ml TGF-β1, TGF-β1 + 0.5 μM cilengitide, or cilengitide. All Western blot quantification data (**b** and **c**) are expressed as mean ± SD after β-tubulin normalization. The experiments on cells were performed in triplicate, with a *n *= 5/group. 2-way ANOVA with Bonferroni’s post-test: *p < 0.05, **p < 0.01, ***p < 0.001

By a cellular point of view, the most detrimental event in cardiac fibrosis is the persistent activation of MyoFB, which causes an excessive deposition of collagen, culminating in the scar formation. This disproportionated increase of ECM, which is markedly stiff, plays a negative role leading to a cronically impaired heart function. Therefore, to better describe the integrin involvement in CF on the basis of different rigidity of growth substrates, Western blot analyses for integrin ανβ5 (Fig. 5a, b) and collagen I (Fig. 5a, c) were performed on total protein extracts of CF seeded on two different substrates and then treated with TGF-β1 and/or cilengitide. The substrates were defined at high stiffness (41 kPa), similar to that of plastic, and low stiffness (30 kPa). The results collected with CF growing on high stiffness were strongly similar to the previous results obtained on plastic support, showing a strong up-regulation of integrin ανβ5 and collagen I protein levels in SHR-CF vs. WKY-CF. Also in this experimental setting, TGF-β1 enhanced the protein expression of both markers as well as cilengitide was effective in counteract the TGF-β1 detrimental effect in both cell lines and able to downregulate the ανβ5 expression more efficiently in SHR-CF than in WKY-CF. On the contrary, the entire system was de-regulated in presence of a lower stiff cell growth substrate. In depth, both CF lines expressed lower amount of integrin and collagen I in comparison with the same cells, growing on a harder stiffness. However, cilengitide was able to downregulate integrin expression in both CF lines also on a lower stiffness. Importantly, despite the lower stiffness of substrate, SHR-CF resulted more responsive to TGF-β1 effect in terms of collagen I, but not of integrin ανβ5, synthesis. Remarkably, cilengitide treatment resulted effective in downregulate also collagen I protein levels.

## Discussion

In this study we found a direct effect of hypertension on integrin-mediated TGF-β1 activation and, by in vitro experiments, the positive effects of cilengitide treatment in decreasing the fibrosis progression by integrin ανβ5 inhibition.

In the last years numerous investigators reported a crucial role played by the integrin-mediated TGF-β1 signaling in ECM remodelling occurring in cardiac fibrosis [30] and the subsequent LVH [31], which are, in turn, strongly associated with hypertension and angiotensin II signaling [32–34].

The first findings of our study revealed a typical perivascular fibrosis in cardiac tissues of hypertensive rat, defining a pro-fibrotic scenario in this animal model. This perivascular fibrosis, strongly consistent with previous investigations [35], was indeed associated with a general hypertension-derived cardiac hypertrophy, underlined by the augmented area of cardiac fibre nuclei [36, 37]. Furthermore, our results were additionally corroborated by a strong up-regulation in cardiac tissue of TGF-β1, key mediator of fibrosis.

All these results on cardiac tissue matched with in vitro evidence: SHR-CF expressed higher levels of α-SMA gene and protein vs. WKY-CF. This led to define these cells as contractile and active MyoFB. Consistently, to date is well-known the detrimental contribution of MyoFB in collagen deposition and ECM remodelling during fibrotic process, leading to irreversible organ dysfunction [28, 38, 39]. Moreover, MyoFB contraction has been shown to directly activate latent TGF-β1, stored in the ECM, by integrin-mediated binding [13, 40]. In fact, it has been extensively reported that fibroblasts, in which α-SMA expression is quite low, are less efficient in the TGF-β1 activation when compared with α-SMA^+^ MyoFB [3, 40]. In light of all these findings, it can be speculated that the hypertensive stimulus, fostered by TGF-β1 treatment, is able to push CF into a pro-fibrotic state, characterized by the up-regulation of TGF-β1/SMAD signaling leading to CF differentiation into MyoFB.

An interesting evidence outlined here is the over-activation of TGF-β1/SMAD signaling in SHR-CF, which is usually maintained in these cells over several cell divisions (data not shown). These findings suggested a chronic activation of the canonical TGF-β1 signaling pathway in CF of hypertensive rats, suggested by the reactivity of starved cells, and further resulting by an increased TGF-β reservoir in SHR heart, determining an autocrine loop. It is really important to point out that, although cultured SHR-CF showed intrinsically enhanced TGF-β1 signaling activation, these cells still strongly responded to exogenous TGF-β1, especially when growing on a substrate with higher stiffness. This evidence indicated that the in vivo hypertensive heart context, more rigid and burdened of latent TGF-β1, rendered SHR-CF more prone and reactive to pro-fibrotic molecular mediators. Among these pro-fibrotic mediators, we can include now also the integrin ανβ5, which displayed in our experimental results higher levels in the SHR heart tissue and in cultured SHR-CF. In 2014, Sarrazy et al. [18] showed, by an in vitro model of human ventricular CF, that the mechanical stimulus (e.g. contraction) activated integrin ανβ5-mediated release of latent TGF-β1. Altogether, these data suggested how hypertension-derived mechanical stress modulated the integrin ανβ5 expression, thereby up-regulating and activating TGF-β1 through its release from the latent complex stored in ECM. Remarkably, our results further draw the attention to the role of TGF-β1, which is boosting the over-expression levels also of integrin ανβ5, especially in SHR-CF.

All these observation drove us to hypothesize the integrin ανβ5 as a putative target for potential pharmacological treatment. Recently, Hatley et al. have extensively analyzed all the available tools, which potentially may play a therapeutic role by acting as αν integrin inhibitors [19]. Among these numerous compounds, we selected cilengitide, because of its deep involvement in pre-clinical and clinical studies [19]. Cilengitide was tested in experimental fibrotic diseases, such as trinitrobenzene sulfonic acid-induced colitis, and its efficacy in inhibiting the fibrosis through the blocking of integrin-mediated activation of latent TGF-β was confirmed both in human and rat intestinal smooth muscles [41]. Moreover, a recent study evaluated the effectiveness of cilengitide in regression of fibrosis on a murine model of systemic sclerosis, a largely known fibrotic systemic disease [42].

In this study, for the first time, we obtained astonishingly evidence on effective inhibition of integrin ανβ5 by cilengitide in our hypertensive-derived in vitro model. The experiments performed allowed us to evaluate different aspects of integrin inhibition. Firstly, the integrin inhibition mediated by cilengitide is necessary and sufficient to decrease the levels of released TGF-β1, which form is able to activate its negative effects. Despite cilengitide does not directly inhibit TGF-β1 but its release, we surprisingly observed that the integrin inhibition provoked a decrease in TGF-β1 pro-fibrotic effects in terms of integrin ανβ5 expression, as well as of collagen I and α-SMA even in presence of TGF-β1. It is important to underline that the effects of cilengitide on WKY-CF are appreciable only as α-SMA protein expression levels. This observation is probably due to the superior efficacy of cilengitide only in presence of higher integrin ανβ5 expression levels. For this reason, cilengitide resulted more effective in SHR-CF than in WKY-CF. The results obtained from the analysis of cells on different stiffness substrates further strengthened our hypothesis, highlighting that this integrin-mediated mechanism is strongly stimulated by a substrate with higher rigidity, as already reported by literature [43–45]. Subsequently, cilengitide action resulted more effective in these conditions. So, in our experiments, treatment with cilengitide positively influenced SHR-CF differentiation into MyoFB, especially on cells growing in ECM with high stiffness and in presence of TGF-β1, decreasing production of pro-fibrotic mediators and α-SMA to levels comparable to untreated WKY-CF.

## Conclusions

Taking into account the numerous important data on pro-fibrotic disease prevention by inhibition of αν integrin family [13, 14, 18], this study may help to define a new role for integrin ανβ5 in CF differentiation into MyoFB, determining this molecule as a novel, considerable, and effective target for the treatment of hypertension-derived cardiac fibrosis. This study sets the stage for essential future in vivo testing of cilengitide in counteracting the detrimental effect of the hypertensive stimulus in cardiac fibrosis development.

## Additional file

**Additional file 1: Figure S1.** Isolated WKY- and SHR-CF equally display vimentin and express mesenchymal markers, confirming their fibroblast nature. (A) Representative images of immunofluorescence for vimentin (in green) on fixed WKY- and SHR-CF (nuclei in blue). Scale bar = 100 μm. (B) Immunophenotype results and analysis on WKY- and SHR-CF for typical mesenchymal (CD90, CD29, CD105), cardiomyocyte (cardiac troponin T, cTnT), endothelial (CD31, CD34), and inflammatory cell (CD45, CD14) markers. FACS results are expressed as mean cell percentage ± SD, *n *= 5/group. Student’s t-test: *p<0.05. **Figure S2.** Dose-response assay on CF treated with different cilengitide concentration. (A) Cell vitality by MTT assay after treatments with three different concentration of cilengitide (0.5, 5, 50 μM). MTT assay quantification data are expressed as mean percentage ± SD. The experiments on cells were performed in triplicate, with a *n *= 3/group. Student’s *t*-test: *p<0.05, **p<0.01. (B) Representative image of MTT assay on 12-multiwell plate. **Figure S3.** Cilengitide inhibition of integrin ανβ5 downregulates laminin protein expression by modulating TGF-β1 gene trascription. (A) Protein expression of laminin in WKY- and SHR-CF after treatments with 5 ng/ml TGF-β1, TGF-β1 + 0.5 μM cilengitide, or cilengitide. Western blot quantification data are expressed as mean ± SD after β-tubulin normalization. (B) Gene expression of TGF-β1 on WKY- and SHR-CF after treatments with 5 ng/ml TGF-β1, TGF-β1 + 0.5μM cilengitide, or cilengitide. qRT-PCR data are expressed as fold ± SD normalized with GAPDH. All the experiments on cells were performed in triplicate, with a *n *= 5/group. 2-way ANOVA with Bonferroni’s post-test: *p<0.05, **p<0.01, ***p<0.001.

## Abbreviations

LVH: left ventricular hypertrophy
TGF-β1: transforming growth factor-β1
CF: cardiac fibroblasts
MyoFB: myofibroblasts
ECM: extracellular matrix
WKY: Wistar Kyoto
SHR: Spontaneously Hypertensive Rat
