# Supplementary material for: Integrin ανβ5 in vitro inhibition limits pro-fibrotic response in cardiac fibroblasts of spontaneously hypertensive rats
Source: J Transl Med. 2018 Dec 12;16:352. doi: 10.1186/s12967-018-1730-1 (PMC6292173; doi:10.1186/s12967-018-1730-1)

**Integrin ανβ5 *in vitro* inhibition limits pro-fibrotic response in cardiac fibroblasts of Spontaneously Hypertensive rats**

Gianluca Lorenzo Perrucci^a,b^*, Veronica Antonietta Barbagallo^b^, Maria Corlianò^b^, Delfina Tosi^c^, Rosaria Santoro^b^, Patrizia Nigro^b^, Paolo Poggio^d^, Gaetano Bulfamante^c^, Federico Lombardi^a,e^, Giulio Pompilio^a,b^

**Additonal Materials**

**Additional Methods**

*FACS analysis*

Immunophenotype analysis of mesenchymal, endothelial, and inflammatory cell markers was performed on CF by using Gallios (Beckman Coulter) flow cytometer. After cell detachment through a nonenzymatic method (TripLE Select, Gibco), cells were resuspended in 100μl of FACS buffer, composed by PBS supplemented with 0.1% BSA (Gibco) and 5mM EDTA (Gibco), and incubated in the dark for 15 minutes with suitable combinations of the monoclonal antibodies and/or isotype-matched control antibodies: CD90-PE, CD29-APC, CD105-APC, CD31-FITC, CD34-FITC, CD45-APC, and CD14-FITC (BD Pharmingen). To evaluate a suitable cardiomyocyte marker, cardiac troponin T (cTnT) was used. To allow anti-cTnT primary antibody (ThermoFisher Scientific) hybridization, CF were firstly fixed and then permeabilized by using BD Cytofix/Cytoperm kit (BD Pharmingen). The secondary antibody used to detect the anti-cTnT was Alexa Fluor® 633 (Life Technology). All samples were washed with 1ml of FACS buffer and centrifuged for 10 minutes at 400 × *g* to remove unbound antibodies. Cells were resuspended in 500μl of FACS buffer and analyzed. CF were gated on a side scatter-forward scatter dot plot to eliminate cell debris. A total of 10,000 events in the CF gated area were acquired. Analyses were performed using the Kaluza software (Beckman Coulter).

*Immunofluorescence for vimentin*

WKY- and SHR-CF were plated on Chamber Slides (Nunc) and placed in growth for 24 hrs with 95% humidity and 5% CO_2_. CF were treated as previously described. Then, slides were rinsed with PBS solution and soaked for about 15 minutes in a solution of 4% PFA. The primary unconjugated antibody raised against vimentin (AbCam) was incubated O/N at 4°C. The goat anti-rabbit IgG secondary antibody conjugated with AlexaFluor488 (ThermoFisher) was incubated for 1 hour at room temperature. As a negative control, species- and isotype-matched IgGs were incubated in place of the primary antibodies. Slides were viewed with Apotome microscope equipped with AxioCam camera (Carl Zeiss) and analyzed with AxioVision 4.7 software (Carl Zeiss).

*MTT assay*

SHR-CF were cultured and treated with 0, 0.5, 5, or 50μM cilengitide for 48 hrs with 95% humidity and 5% CO_2_. The growth medium was replaced with a solution of 10% 5mg/ml of 3-(4,5-dimethylthiazol-2-yl)-2,5-diphenyltetrazolium bromide (Sigma-Aldrich) (MTT dye) in PBS and 90% Opti-MEM medium (Gibco) for 3 hrs with 95% humidity and 5% CO_2_. The MTT-Opti-MEM medium was finally replaced with dimethyl sulfoxide (DMSO) for 10 minutes at room temperature and the multiwell plate was read at 590nm and 620 nm by multiwell spectrophotometer Mithras LB 940 (Berthold Technologies).

**Additional Figure legends**

**Additional Figure S1 - Isolated WKY- and SHR-CF equally display vimentin and express mesenchymal markers, confirming their fibroblast nature.** (A) Representative images of immunofluorescence for vimentin (in green) on fixed WKY- and SHR-CF (nuclei in blue). Scale bar = 100μm. (B) Immunophenotype results and analysis on WKY- and SHR-CF for typical mesenchymal (CD90, CD29, CD105), cardiomyocyte (cardiac troponin T, cTnT), endothelial (CD31, CD34), and inflammatory cell (CD45, CD14) markers. FACS results are expressed as mean cell percentage ± SD, *n*=5/group. Student’s *t*-test: **p*<0.05.


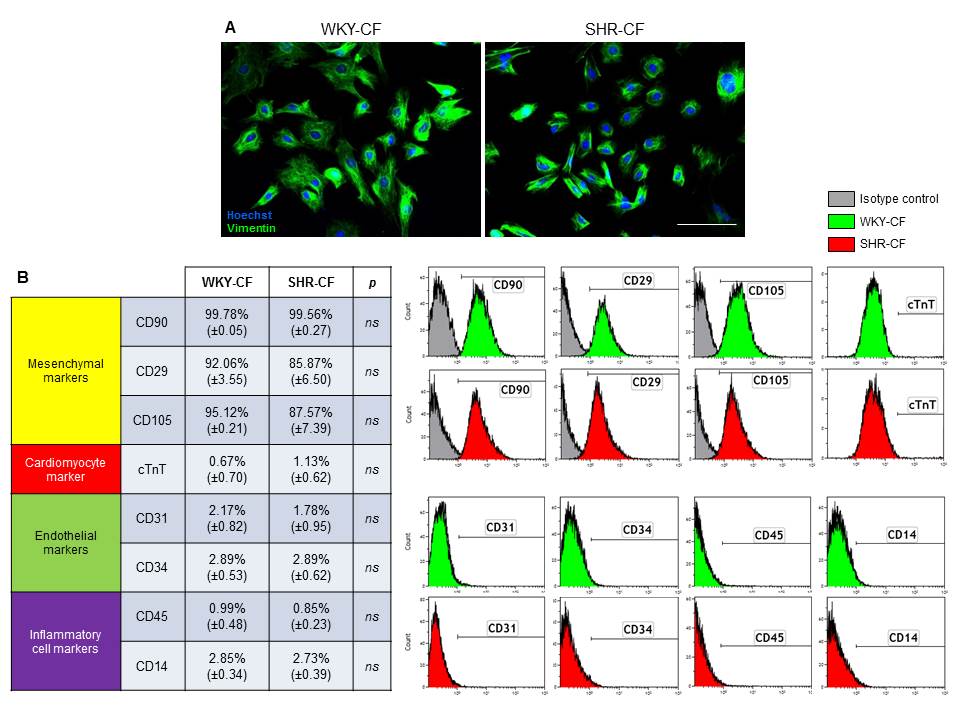


**Additional Figure S2 - Dose-response assay on CF treated with different cilengitide concentration.** (A) Cell vitality by MTT assay after treatments with three different concentration of cilengitide
(0.5, 5, 50μM). MTT assay quantification data are expressed as mean percentage ± SD. The experiments on cells were performed in triplicate, with a *n*=3/group. Student’s *t*-test: **p*<0.05, ***p*<0.01. (B) Representative image of MTT assay on 12-multiwell plate.


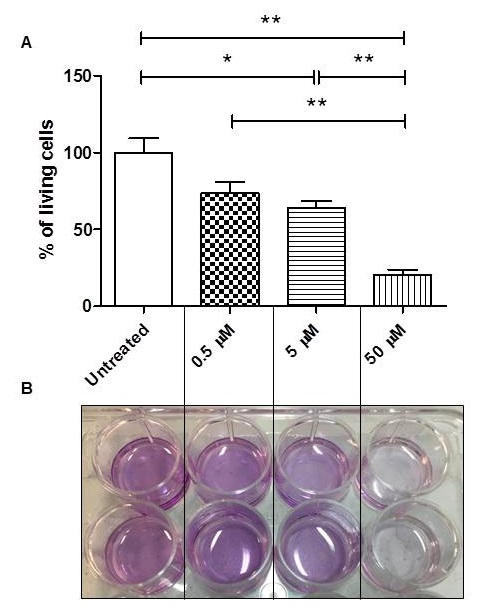


**Additional Figure S3 - Cilengitide inhibition of integrin ανβ5 downregulates laminin protein expression by modulating TGF-β1 gene trascription.** (A) Protein expression of laminin in WKY- and SHR-CF after treatments with 5ng/ml TGF-β1, TGF-β1 + 0.5μM cilengitide, or cilengitide. Western blot quantification data are expressed as mean ± SD after β-tubulin normalization. (B) Gene expression of TGF-β1 on WKY- and SHR-CF after treatments with 5ng/ml TGF-β1, TGF-β1 + 0.5μM cilengitide, or cilengitide. qRT-PCR data are expressed as fold ± SD normalized with GAPDH. All the experiments on cells were performed in triplicate, with a *n*=5/group. 2-way ANOVA with Bonferroni’s post-test: **p*<0.05, ***p*<0.01, ****p*<0.001.


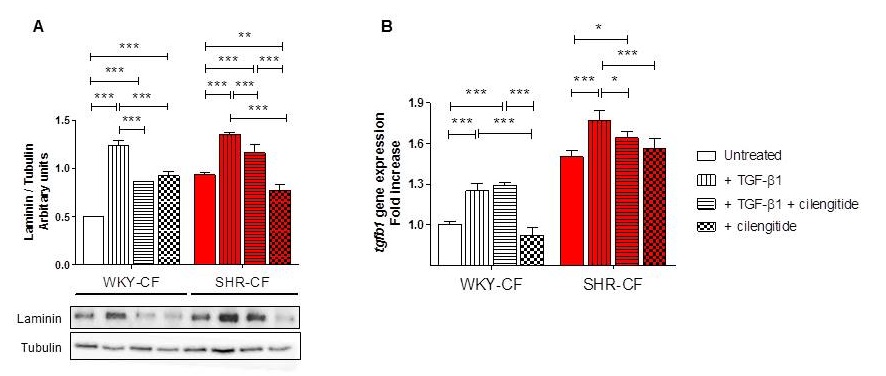

Supplement: Supplementary file 1 — Additional file 1: Figure S1. Isolated WKY- and SHR-CF equally display vimentin and express mesenchymal markers, confirming their fibroblast nature. (A) Representative images of immunofluorescence for vimentin (in green) on fixed WKY- and SHR-CF (nuclei in blue). Scale bar = 100 μm. (B) Immunophenotype results and analysis on WKY- and SHR-CF for typical mesenchymal (CD90, CD29, CD105), cardiomyocyte (cardiac troponin T, cTnT), endothelial (CD31, CD34), and inflammatory cell (CD45, CD14) markers. FACS results are expressed as mean cell percentage ± SD, n = 5/group. Student’s t-test: *p<0.05. Figure S2. Dose-response assay on CF treated with different cilengitide concentration. (A) Cell vitality by MTT assay after treatments with three different concentration of cilengitide (0.5, 5, 50 μM). MTT assay quantification data are expressed as mean percentage ± SD. The experiments on cells were performed in triplicate, with a n = 3/group. Student’s t-test: *p<0.05, **p<0.01. (B) Representative image of MTT assay on 12-multiwell plate. Figure S3. Cilengitide inhibition of integrin ανβ5 downregulates laminin protein expression by modulating TGF-β1 gene trascription. (A) Protein expression of laminin in WKY- and SHR-CF after treatments with 5 ng/ml TGF-β1, TGF-β1 + 0.5 μM cilengitide, or cilengitide. Western blot quantification data are expressed as mean ± SD after β-tubulin normalization. (B) Gene expression of TGF-β1 on WKY- and SHR-CF after treatments with 5 ng/ml TGF-β1, TGF-β1 + 0.5μM cilengitide, or cilengitide. qRT-PCR data are expressed as fold ± SD normalized with GAPDH. All the experiments on cells were performed in triplicate, with a n = 5/group. 2-way ANOVA with Bonferroni’s post-test: *p<0.05, **p<0.01, ***p<0.001. [file 12967_2018_1730_MOESM1_ESM.docx]
